# Sustainability issues of commercial non-timber forest product extraction in West Suriname

**DOI:** 10.1186/s13002-018-0244-5

**Published:** 2018-06-28

**Authors:** Tim van den Boog, Janette Bulkan, James Tansey, Tinde R. van Andel

**Affiliations:** 1 Forest Resources Management, Faculty of Forestry, University of British Columbia, Vancouver, Canada; 2 Sauder School of Business, University of British Columbia, Vancouver, Canada; 3 Naturalis Biodiversity Center, Vondellaan 55, 2332 AA Leiden, Netherlands

**Keywords:** Indigenous peoples, Globalisation, Tenure security, Suriname, Stingray (*Potamotrygon boesemani*)

## Abstract

**Background:**

Non-timber forest products (NTFPs) have been traded for millennia by indigenous communities. Current increased demands driven by globalisation, however, put more pressure on local harvesters and their surrounding ecosystems. The safeguarding of indigenous access rights to harvesting grounds is needed, either through communal land titles or collaborative management agreements, both to secure prior indigenous rights and to minimise further negative ecological impacts.

**Methods:**

This study was carried out in two indigenous communities in West Suriname located along the Corentyne River. We assessed the three economically most important NTFPs for each community. We determined the land tenure status of harvesting grounds and negative impacts on target species and/or ecosystem. Ethnobotanical data were collected (*n* = 53), and semi-structured interviews were held with hunters and gatherers (*n* = 13). Local and national maps were acquired, and their data merged.

**Results:**

Results showed that the communities have no tenure security over their most important harvesting sites. These collection sites are State owned and some under (active) logging concession. All of the traded wild animal populations had decreased because of increased local and non-local commercial interest, especially the stingray *Potamotrygon boesemani* (first described in 2008), which was traded for US$250 per live specimen. The stingray population had become imperilled within months as local and (inter-) national regulations for this species are non-existent.

**Conclusions:**

We stress the urgent need for collaborative management agreements over the harvesting sites between the government of Suriname and the indigenous communities to prevent further non-local developments and harvesting to disturb the local economy. An immediate moratorium on the export of *P. boesemani* is necessary to prevent the extinction of this endemic stingray.

## Background

Non-timber forest products (NTFPs) are important resources to sustain the livelihoods of many rural communities. A vast range of NTFPs is being sold on local, national and international markets, with annual profits of billions of US dollars [1]. Internationally marketed NTFPs range from wild animals for traditional remedies, exotic foods or the pet trade [2, 3] to a wide variety of medicinal plants [4] and natural cosmetic products. Commercialisation of NTFPs plays an important role in mitigating poverty of local harvesters and processors, but can also enrich middlemen at the expense of the primary harvesters and local processors [5, 6].

Every commercialised NTFP has a production-to-consumption route or value chain [5], linking the economics of harvesting, processing and transporting products to final consumers [7]. The involved actors range from individual harvesters and accomplices to middlemen and large-scale commercial factories [5]. Short commodity chains, locally sold NTFPs for instance, are simple and involve few, if any, controls or regulations [8]. Internationally marketed forest products, however, have a much more complex producer-to-consumer system with many more actors involved and can be subject to (inter-)national trading regulations [5].

The worldwide trade of some NTFPs are managed by regulatory frameworks [9], created by governments and international institutions, such as the United Nations Food and Agriculture Organisation (UN-FAO), the Convention on Biological Diversity (CBD), the World Bank, the Convention on International Trade in Endangered Trade of Wild Fauna and Flora (CITES), the International Union for Conservation of Nature (IUCN) and the Trade Record Analysis of Flora and Fauna in Commerce (TRAFFIC). However, many wild plants and animals are still not covered by international frameworks and can therefore be subject to overexploitation. Other conventions and declarations, such as the International Labour Organization Convention 169 (ILO 169) and the United Nations Declaration on the Rights of Indigenous Peoples (UNDRIP), address the need for safeguarding the customary lands, territories, resources and rights of indigenous peoples. Legal rights over lands and resources are often not held by the communities that utilise them, although, in some cases, they need formal access and extraction rights to the land and its resources in order to collect forest products [10].

When the demand for NTFPs increases heavily, rural communities are often driven to over-exploit them, either because of opportunism, economic insecurity, and lack of management rules or secure property rights [1]. Consequently, harvesting practices can have negative ecological impacts. Entire species’ populations can become endangered [3, 11], while by-catch and destructive harvesting techniques can lead to unstable target population dynamics, ecosystem imbalance and habitat destruction [1]. The commercialisation of NTFPs should therefore focus not only on market access, value-adding, poverty alleviation, or producer-to-consumer chains but also on the negative ecological and population impacts that can result from over-harvesting or destructive harvesting practices.

Suriname lies on the eastern edge of the Guiana Shield which is located in the northeast corner of South America, lying for the most part between the Orinoco and Amazon River basins, to the west and south respectively. The Guiana Shield, together with the Brazilian and West African shields, is considered one of the planet’s oldest land surfaces (Gibbs and Barron 1993). Not surprisingly, many of the trees and other plants which can survive and grow on its infertile soils are specialised and considered endemic to the Guiana Shield.

Suriname has retained a tropical forest cover of over 90% and harbours a great diversity in flora and fauna. Four indigenous and six tribal peoples inhabit the interior forests, with an estimated population of 12,000 and 60,000, respectively [12]. Tribal peoples, also known as Maroons gained international recognition in 1993 with equivalence to indigenous peoples for the law [13]. The country acceded to CITES in 1980, ratified the American Convention on Human Rights in 1986 and the CBD in 1996 and adopted UNDRIP in 2007. Many wild plants and animals are commercialised on both the national and international market [4, 14, 15]. The export of living birds from Suriname is the most profitable, followed by reptiles and amphibians, altogether worth an estimated US$473,000 in 2006 [16]. In the same year, ca. 136 tonnes of medicinal plants were sold on the domestic market with an estimated value of US$1,123,000 [4]. Exported medicinal plants to the Netherlands, its former colonial ruler, have an estimated value of US$453,180 annually [4]. However, few recent data are available for the NTFP trade and its ecological impact.

In Suriname, only 50,000 ha of forests are legally titled as privately owned; all remaining forests are under State jurisdiction [17]. In 1992, the category ‘communal forests’ was introduced in the Forest Management Act as ‘...forests for the benefit of forest peoples living in villages and settlements in tribal societies, and that serve to meet subsistence needs of food and forest products, as well as for the purpose of possible commercial timber extraction, the collection of Non-Timber Forest Products, and land clearing for agricultural use’ [18]. In reality, communal forests only cover small parts of indigenous and tribal peoples’ (ITPs) customary territories and do not correspond to the demarcation maps made by local communities. Furthermore, the government of Suriname has allocated customary territories to mining and logging companies, thereby reducing ITPs’ access to lands and resources [18–21]. Suriname’s ITPs have brought cases detailing their land rights claims against their government before national courts and the Inter American Court of Human Rights (IACHR). Suriname was found guilty by the IACHR of failing to legally recognise the collective land rights of the Saramaka tribal people in 2007 and of the Kaliña and Lokono indigenous communities in 2015 [22, 23]. Although the IACHR’s rulings are binding, the court itself lacks an enforcement mechanism. To date (January 2018), the government has failed to implement the judgements of the IACHR.

This paper is based on research carried out in two indigenous communities in West Suriname in 2016: the predominantly Arawak community of Apoera and the Trio community of Sandlanding [24]. Our aim was to identify the major commercialised NTFPs, their corresponding production-to-consumption chains, and find out whether harvesting practices were potentially unsustainable. Additionally, an assessment was done on the status of the communities’ land rights. The following research questions were assessed:

1. Which NTFPs are most important for the communities’ economic welfare?
2. Are there indications that commercial NTFP harvesting has a negative impact on either the species or the local ecosystem?
3. What is the tenure status of the lands from which these NTFPs are harvested?

## Methods

### Study sites

This research took place in Apoera (5° 11.43′ N and 57° 10.38′ W) and Sandlanding (5° 9.81′ N and 57° 10.20′ W), which falls under the jurisdiction of Apoera [24]. This location was selected for its unique composition of indigenous communities and because few studies have been carried out in this region. Both villages are located on the right bank of the Corentyne River, which marks the border between Guyana and Suriname. The area has a tropical rainforest climate with a mean annual temperature of 27 °C and an annual precipitation of 1895 mm. To get to the closest larger towns entails a 7 to 10-h vehicle trip east towards the capital Paramaribo, or a 120-km boat ride north to Nickerie.

### Ethnobotanical data collection

Prior to fieldwork, a meeting was set up with the local authorities of Apoera and Sandlanding to request their Free, Prior and Informed Consent (FPIC) to the proposed research project, in accordance with the stipulated guidelines [25]. This research project was covered by UBC ethics certificate number H15-02527. Signed FPIC agreements were also a prerequisite for the permit issued by the Surinamese Forest Management and Production Control (SBB, equivalent to a Forestry Commission). Fieldwork was done between March and April 2016 for 5 weeks. Forest walks were held with 13 Trio and Arawak informants (male and female) who were singled out by village authorities for their ethnobotanical knowledge. Walks usually started early in the morning and lasted for about 4 h at collection sites that the informants picked. Some informants preferred to share information while collecting NTFPs; others solely focused on sharing their local names and uses. Voucher specimens were collected, identified and deposited in the National Herbarium of Suriname (BBS) and Naturalis Biodiversity Center (L) at Leiden, the Netherlands. NTFPs without voucher material were identified by means of photographs, local names, and recent literature on NTFP from the Guianas [26–28]. Scientific names were verified through theplantlist.org.

### Interviews

The economically most important NTFP species (plants and animals) were identified by means of semi-structured interviews held with seven hunters and NTFP gatherers from Apoera and six from Sandlanding. Interview questions were based on the national socioeconomic surveys in forestry, modules A, B and C, developed by the Food and Agriculture Organization (FAO), Center for International Forestry Research (CIFOR), International Forestry Resources and Institutions (IFRI) and the World Bank [29]. Participants were asked to list the three most important commercialised wild plants and animals, following the FAO et al. (2016) socioeconomic survey guidelines. We collected information on where and when those NTFPs were harvested, their processing methods, and to whom they were sold, in order to make a profile of the products’ value-chains. The questionnaire also covered the units and pricing of the main commercial NTFPs and included questions on the legal ownership status of and access to the lands where these NTFPs were harvested. We asked whether the availability of the most important NTFPs had recently increased or declined and the reasons for such changes. Additionally, market surveys were held on three occasions, during which vendors were questioned about collected NTFPs. Finally, middlemen and international traders were contacted by phone or email to track the movements of the NTFP exports from West Suriname.

## Results

### Land rights in West Suriname

**Fig. 1:**
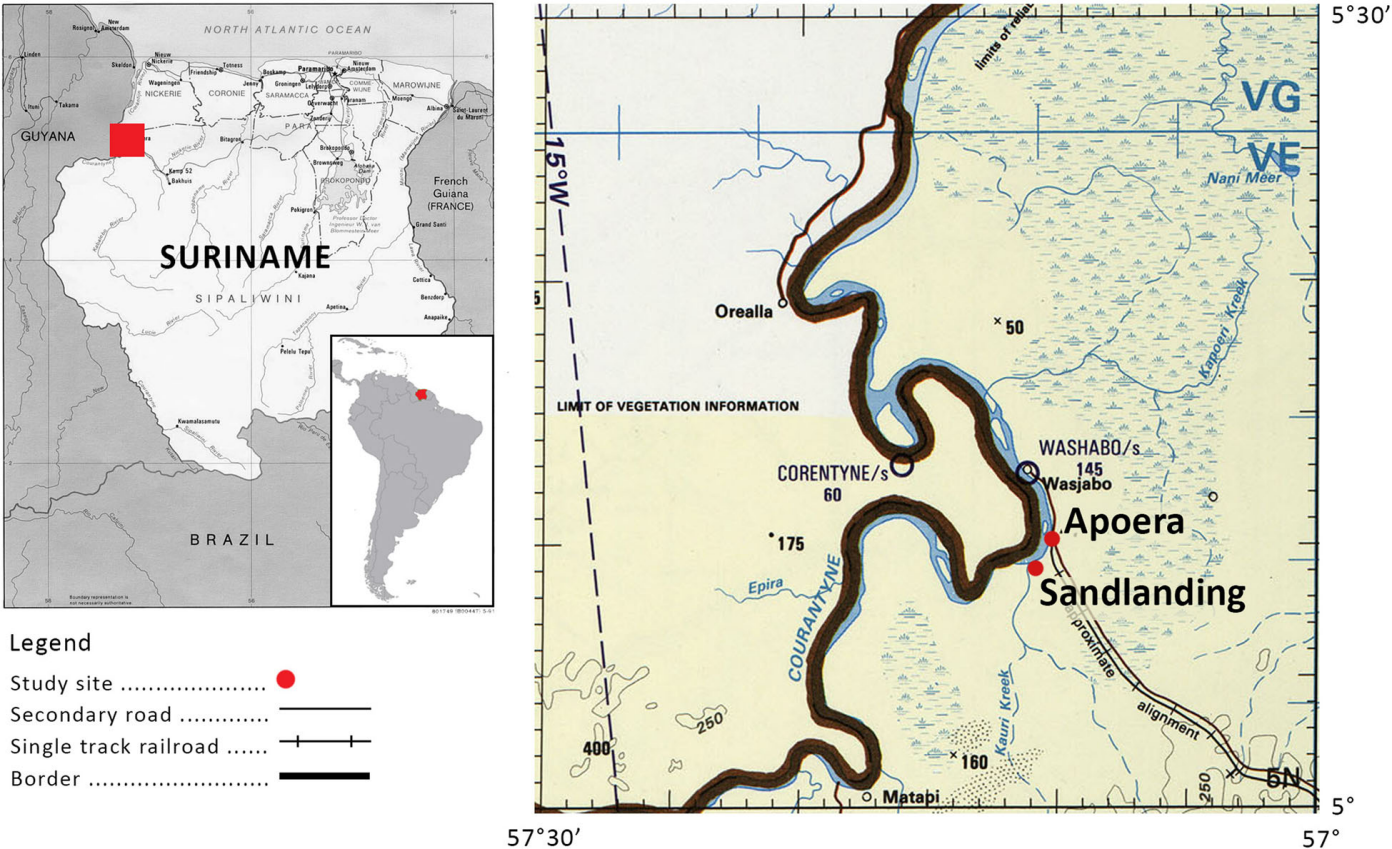
Study sites Apoera and Sandlanding along the Corentyne River. Modified from http://www.lib.utexas.edu/maps/tpc/txu-pclmaps-oclc-22834566_l-28a.jpg 8

**Fig. 2:**
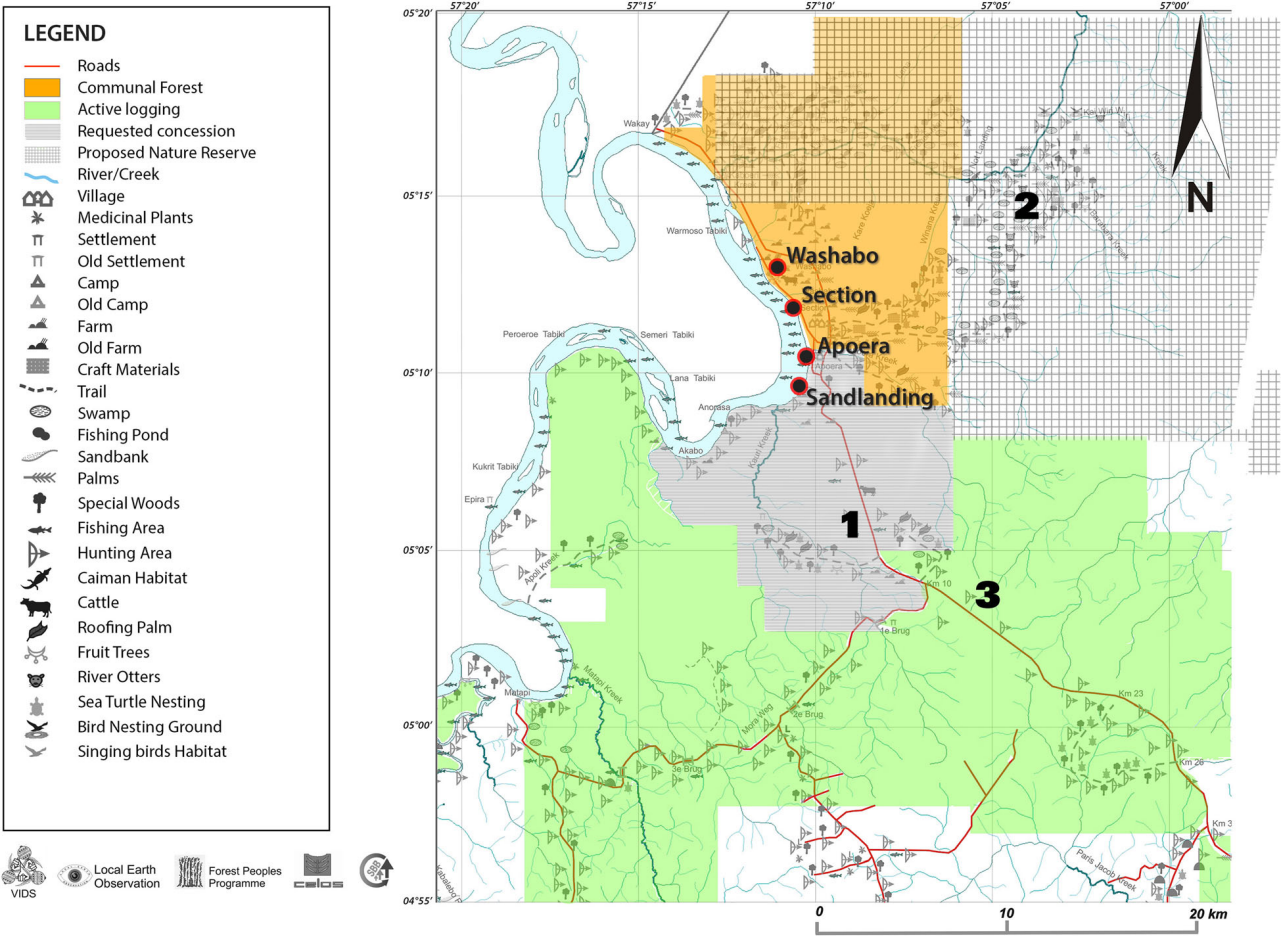
Map of West Suriname, visualising customary uses, communal forest (in orange) and active logging concessions (in green). Numbers 1, 2 and 3 indicate important NTFP location sites

The villages of Apoera, Section and Washabo (Fig. 1) had been assigned one collective logging concession area by the government in the 1940s, then re-designated in 1992 as a communal forest (Fig. 2, orange). Up to 1997 a single traditional captain or village leader held administrative responsibility for all three villages. Since then, each village has been administered by separate traditional authorities. Currently, the highest authority in Apoera is a captain who is assisted by six *basyas*, three women and three men. A *basya* is one rank lower than captain in the traditional authority system and can be seen as an advisor or assistant to the captain. The highest traditional authority of the Trio in Sandlanding, a satellite community of Apoera, is a *basya*. Within the communal forest, the villagers are allowed to practice their traditional customs, such as hunting, farming, logging and gathering of NTFPs, including for market sales. However, as noted earlier, this communal forest covers only a small portion of the territory that is customarily used and ancestrally claimed by the Arawak indigenous communities. Customary uses are represented by different icons in Fig. 2.

The area where the Trio live, Sandlanding, and hunt falls outside the communal forest, but within the customary territory of Apoera, to which they gained access and use rights in 2001 after successful negotiations with the Apoera authorities. The latter allowed the Trio to build a settlement and to hunt in the area. However, statutorily, these lands are State-owned and currently under official request for expanded logging and mining activities. In Fig. 2, a map of West Suriname displays customary uses, the designated communal forest and active logging concessions documented by SBB. These were mapped by community members in a collaborative project with the Association of Indigenous Village Leaders (VIDS), a national Indigenous Peoples Organisation (IPO). For the full customary territory map, please consult van den Boog et al. [24]. Active natural resources concession areas, shown in green, overlap customary territories and disrupt the traditional activities of the indigenous communities of West Suriname.

### Commercialised plant NTFPs

Appendix 1 provides a complete list of commercial NTFPs from plant and animal origin. In total, seven wild plant species were commercialised in the study area, either traded on the local market or to middlemen. In Apoera, crabwood oil derived from the seeds of *Carapa guianensis* Aubl. was the most important plant NTFP, followed by Brazil nuts (*Bertholletia excelsa* Bonpl.) and sawari nuts (*Caryocar nuciferum* L.).

Crabwood seeds were mainly collected in an area outside the communal forest currently under request for allocation of logging licences (Fig. 2, number 1). The seeds are collected as close as possible to roads because a filled bag weighs over 40 kg. The crabwood tree belongs to the mahogany family and is similarly highly prized for furniture and flooring. These tree stands would be easily accessible to loggers if logging concession licence(s) were issued. Crabwood trees closer to Apoera had been removed without the consent of the community when an airstrip was built ca. 10 years ago. This illustrates the community’s lack of tenure security over the customary lands in which they harvest crabwood nuts. Repeated seed collection in the same area is likely to reduce the number of seedlings that support the regeneration of crabwood populations. Excessive seed removal by human harvesters may also jeopardise the animals that feed on them. However, since the surrounding forest ecosystem is relatively pristine, animals are likely to forage in different places, minimising the additional negative impacts of foraging humans.

The extraction of oil from the nuts from the crabwood tree involves hard manual work and a lengthy process. The nuts are collected from the forest floor, boiled, dried, left to rot for a few weeks and cracked open to obtain the inner seed paste, which is then placed in bowls and exposed to the sun to allow the heated oil to drip out (see Appendix 2). Crabwood oil is used as a cosmetic—externally applied on dry skin and hair—and medicinally when swallowed as a laxative. In 2016, middlemen from Paramaribo and Nickerie paid US$5–7 per litre of crabwood oil at the Apoera market. Some relatives of crabwood oil producers sold the product in the Paramaribo markets to capture a larger share of the accruing value. In 2017, a bottle of 125 ml crabwood oil cost about US$10 on amazon.com, or up to 16 times the price paid in Apoera market.

Brazil nuts, the second most commercialised NTFP in this area of west Suriname, are well-known in the developed world. The fruit capsule takes over 12 months to ripen, after which it drops on the ground. The nuts are collected from February through May. The outer shell is extremely tough and needs to be opened with a machete. Once opened, many small hard-shelled nuts are revealed. These are valued for consumption and to extract an oil with many uses [30]. Brazil nuts were sold on the local market for US$1.80/kg and were bought by the bag by a local tradesman for resale in Paramaribo. Online, these nuts are sold for about US$25–35/kg, a 14- to 19-fold increase over the local price.

The third most important NTFP, *Caryocar nuciferum* nuts, were locally sold at the same price, but collecting takes place in June and July. *C. nuciferum* nuts are mainly used for nourishment. Both nut species were collected outside the communal forest on State land (Fig. 2, number 2). As this part of the communities’ customary territory is located far from accessible roads and in quite swampy terrain, it is unlikely that disturbance from concessions would occur soon. This area was proposed to be set aside as a nature reserve in 1979 by the government and is still so labelled on the forest tenure map (SBB, 2013). A document signed by the statutory district commissioner Hr. Arichero and local village captain G. Mc-Intosh on 14 October 1979 (seen and photographed by the first author) states that indigenous communities are allowed to practice traditional activities within the reserve for subsistence use only, which excludes NTFP gathering for commercial purposes if this area were to be declared a nature reserve. It also states that the indigenous communities have to thrive to become similar to ‘Surinamese citizens’.

In Sandlanding, the only plant products harvested by the Trio villagers for trade were seeds of *Ormosia costulata* (Miq.) Kleinhoonte to make jewellery. These attractive small seeds are orange to red with a purple to black spot. Trio women sold their jewellery usually every other Saturday at the local market in Apoera, a 40-min walk. They explained that locals did not frequently buy jewellery, but the few national and international tourists would. The Trio women kept on making jewellery even while they had no access to larger markets because of the distance and a lack of middlemen. In March–April 2016 they had hundreds of pieces of jewellery in stock. Through a recently-established connection in Paramaribo, they now sell jewellery in the capital and in shops in the Netherlands. *Ormosia* trees produce many seeds, which are not all collected. The Trio reported in 2017 that in the area where they collected seeds, a new ‘flag-line’ was set out by the logging company (Fig. 2, number 3), which denoted the new border of near-future logging activities in this part of Apoera’s customary territory. Soon, the Trio will have to find another site to collect this NTFP.

### Commercialised animal NTFPs: Potamotrygon boesemani

For both communities, a stingray endemic to the Corentyne River, *Potamotrygon boesemani*, was by far the most important commercially traded NTFP in 2016 (Appendix 1). There is little literature on this remarkable fish, which was described as a new species in 2008 [31]. Known locally as ‘spari’, this stingray is not yet listed on the IUCN Red List of threatened species. Spari, which is well known to the indigenous communities for its venomous sting and its delicious taste, became a major commercial NTFP only in 2015. Formerly, it used to be cooked by the Trio and eaten as a delicacy. However, since living boesemani stingrays have become a highly desirable showpiece in aquaria worldwide, buyers from the city paid up to US$300 per live specimen in 2016. With the generated income, the Sandlanding Trio, who only gained access to electricity in March 2016, were buying freezers and large flat screen televisions and starting to replace some traditional wooden houses for partially concrete ones.

The stingrays were most easily caught at night during the dry season, when the water level in the river and creeks was lower. Several methods were used to catch the stingrays. Spears with a small, sharp tip were shot through the animal’s fins to immobilise it, or Y-shaped sticks were used to pin the stingrays to the ground. Other fishermen used nets to trawl the river and creek beds. Once a stingray was immobilised, fishermen wearing swim goggles cut off the animal’s stings while underwater using scissors or a knife. Cutting off a ray’s sting is bad practice as it is a sensitive part of the animal and it could cause infections. This species has one or two serrated caudal stings with a length up to 2.5 times the tail width. People who are stung in their feet or legs suffer extreme pain for weeks, although the sting is not fatal when the right antidote is available. According to the fishermen, a sting in the upper body could be fatal, although this did not happen during fieldwork.

Transportation of the living stingrays was very difficult. Local fishermen reported that a boesemani stingray needed fresh, oxygenated water every 15 to 30 min, otherwise it would suffocate. Transportation was most successful in cages trailed behind a boat at slow speed or in baskets with air pumps. However, since most fishermen did not own a boat, air pumps, and cages or lacked the knowledge about how to keep this animal alive in captivity, we estimated that hundreds of stingrays died during transport to the villages between January and April 2016. People who did not own a boat would go fishing at night, wading with rubber boots through creeks with a headlamp, a spear in one hand, and an empty basket in the other and surrounded by spari hunters in small boats. In both directions of the creek, headlamps from stingray hunters would be visible. Most villagers, including women who generally did not hunt, joined in the search for stingrays. All those without a daily job who could get their hands on a boat, would go for days or week-long trips to search for stingrays. In February 2016, the animals were still found close to the villages, but populations soon started to decline so that hunters were travelling further and further away. In late March and April 2016, people often did not succeed in catching a single specimen on multiple day hunts, even as far away as Wonotobo falls, 7 h upriver by small motorboat. Similar activities took place on the left bank, the Guyanese side, of the Corentyne River.

**Fig. 3:**
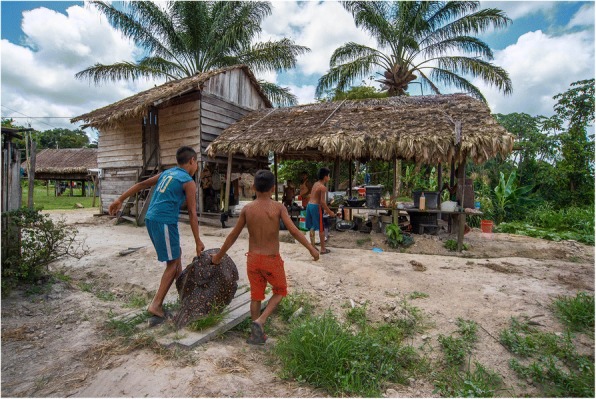
A large boesemani stingray that died prior to arrival in the village was boiled and fed to the dogs in Sandlanding

Buyers from Paramaribo arrived in charter planes, by car or in four-wheel drive vans and buses. Guyanese buyers tried to buy specimens from fishermen on the river before the animals were landed on the Surinamese riverbank. In Apoera, a middleman set up three large tanks equipped with air pumps to store living stingrays. In Sandlanding, an inflatable swimming pool was used to store some specimens. Buyers would delay their arrival until larger batches had come in (up to 15 or 20 stingrays at a time). Many of the stingrays, however, died before arrival in the village (Fig. 3). On a single trip to the Wonotobo Falls, 15 animals died, worth US$3750, or the equivalent of 2678 Trio *Ormosia costulata* bracelets. Transport to the capital by car or bus posed further risks, as many stingrays died during the journeys by road. One buyer reported that a live stingray could fetch US$1250, five times the price paid in the village, when sold to an international buyer. Mid-sized (18–25 cm) females were the most wanted. Following the increasing popularity, the single government official with authority to issue export permits increased the tax per specimen from US$25 in 2012, to US$100 in 2015 and to US$200 in 2016. The Surinamese authorities did not want to disclose any export data on *P. boesemani*.

**Fig. 4:**
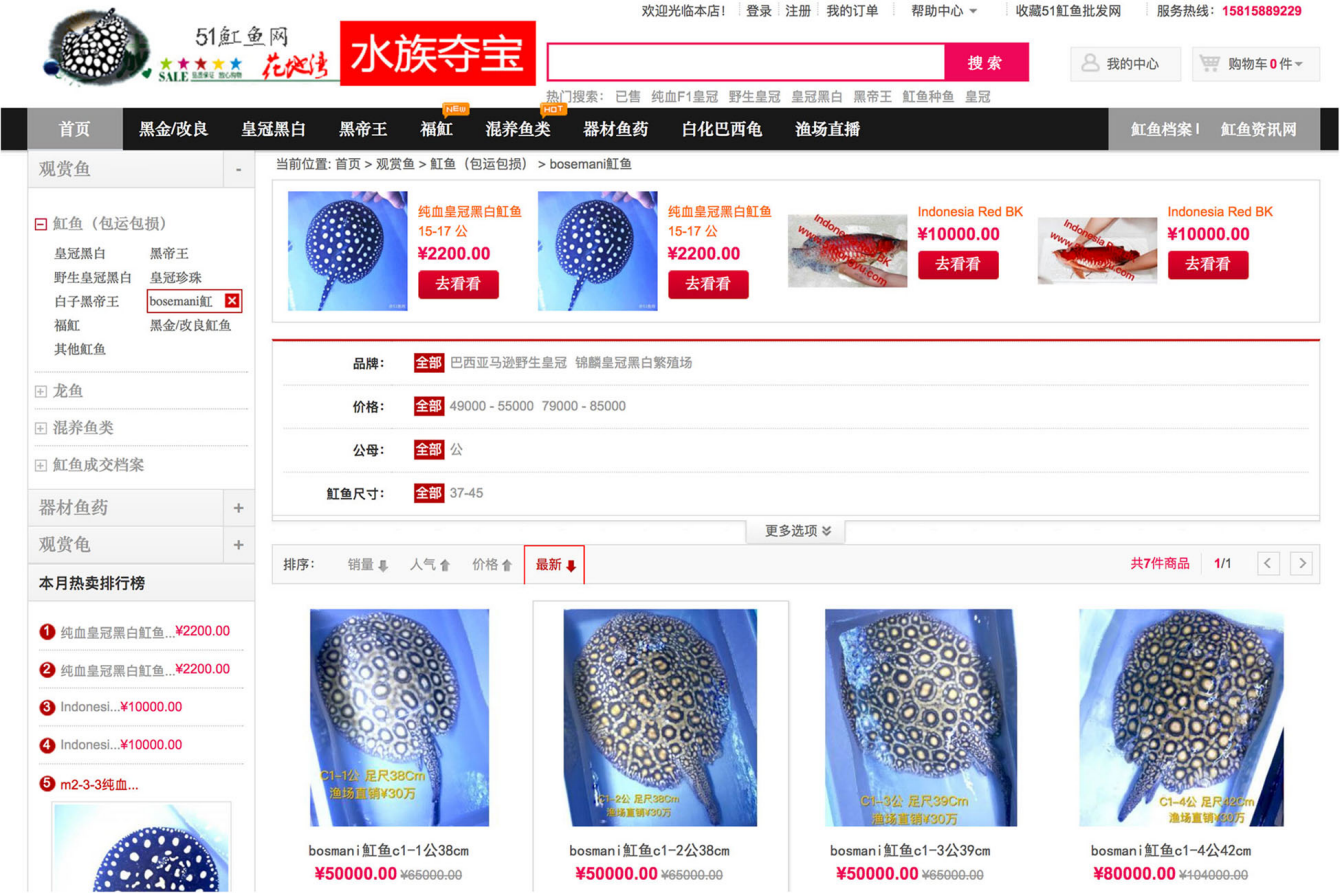
Potamotrygon boesemani online for sale in 2017 on a Chinese website (http://51hongyu.com) for up to 80,000 yuan (ca. US$ 12,100)

**Fig. 5:**
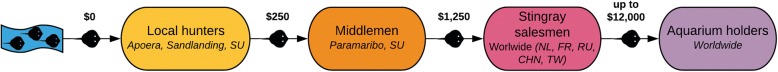
Value chain in US$ of *Potamotrygon boesemani*

When we contacted international stingray traders to track export routes, we found spari specimens for sale in France, the Netherlands, Russia, China and Taiwan. The most expensive specimen was found in China (sold under the names stingray, 魟; boesemani, 波斯玛尼) for about US$12,100 for a 42-cm sized male (Fig. 4), 46 times the amount paid to local fishermen. Apparently, international transport also entailed high mortality risks, as a Taiwanese dealer explained: ‘I got Boesemani rays from Suriname directly. This ray is the most difficult [animal] for acclimation I have ever met. Really wild style rays, and I lost many pieces’. His retail price was US$10,000 for a 30–40 cm sized pair, which included the US$400 paid per specimen for air transport to Taiwan. The boesemani stingrays are often sold in pairs. On the Dutch market, prices were lower: US$3200 for a pair of 15–25 cm, and US$ 8500 for a 45–50 cm sized pair. For an overview of the value of boesemani rays in the entire value chain, see Fig. 5.

In the de facto open access Corentyne River area with no regulations on offtake, local fishermen opt to take all the ‘swimming cash’ they can. Theoretically, the stingrays are common-pool resources, belonging to the Surinamese Nation-State. The exclusion of non-local fishermen or regulation of off-take by the governmental authority is therefore possible, however costly [32]. In practice, the Corentyne watershed operates under an open access regime so that the off-take of any commercialised fish species can only be sustainable when the demand is low or the price is uneconomic and harvesting practices do not harm the populations and the surrounding ecosystem [33].

Since *Potamotrygon boesemani* was only recently scientifically described, its role in the ecosystem of the Corentyne River basin is still unclear. Research on different stingray species shows that they are an ecological keystone species that greatly influence their ecosystem as they modify physical and biological habitat elements through foraging and predation [34]. Moreover, stingrays are known to prey on different trophic groups [35]. Removing such a substantial number of boesemani stingrays within a few months is therefore likely to have serious impacts on trophic systems of the Corentyne River basin. The species’ narrowly endemic distribution, the great losses during transportation and the lack of any (inter-)national regulatory regime to protect this species in combination with a sudden increase in international demand suggest that the boesemani stingray is likely to become extinct in the Corentyne River.

#### Other economically important animal species

The white-lipped peccary (*Tayassu pecari*) is the second most important commercial animal NTFP for the communities of Apoera and Sandlanding (Appendix 1). This species is IUCN red-listed as vulnerable (VU) because of an estimated decline of 30% of the population within the last 18 years due to (illegal) hunting and habitat loss [36]. By Surinamese statutory hunting laws, this animal is only allowed to be hunted and captured (and sold) during the open season from August through to March, with a maximum of one specimen per trip [37]. Indigenous communities in the northern half of the country are also required to abide by these laws. These regulations are also locally enforced by appointed officers of SBB and police.

The lowland paca (*Cuniculus paca*) is listed by the IUCN as LC, ‘Least Concern’. For Apoera’s community, this is the third most important commercial animal NTFP. Members of the Trio community do consume and sell paca as well, but it did not appear in their top three. Paca are bought directly from hunters by locals for subsistence purposes for ca. US$2.80/kg. Outsiders from mainly Nickerie and Paramaribo come to West Suriname to buy or hunt these animals themselves. Local hunters reported that almost all bush meat populations are in decline, especially when compared to 20 years ago and longer. The reasons they gave for the decline were the following: (1) increased demand for bush meat for subsistence by local people and outsiders; (2) increased collection of bush meat for local sale; (3) game animals keep their distance from nearby natural resources concession areas (machinery noise, people); (4) animals learn to avoid hunters.

The Apoera and Sandlanding villagers hunt entirely outside their communal forest, as indicated by the bow-and-arrow icons on the map in Fig. 2. Expansion of logging activities closer to the villages would have negative consequences for both commercial and subsistence hunters.

#### Wolf fish (*Hoplias aimara*)

The wolf fish is a large predator that reaches lengths of over 100 cm [38]. This species is more commonly consumed and sold by the Trio than their neighbours from Apoera, likely because it occurs in their former settlement near the Wonotobo falls where they still fish. They placed this animal as their third most important commercial NTFP and sold it for US$2.40 per kg. They used to sell their catch on the day of their return from their fishing trip, because Sandlanding was not connected to any electrical power grid until March 2016. Consequently, villagers did not own fridges and freezers. Both local and city people were frequent buyers. The Trio community noted a decrease in the occurrence of the wolf fish in the Corentyne river. Besides overfishing from locals, they reported an increase in tourist fishing trips that go far up the river to catch wolf fish.

## Discussion

### Widely distributed versus endemic species

The challenges to sustainable offtake of the three most important commercialised plant NTFPs are different to the ones confronting faunal species in this area of north western Suriname. There are two key differences—one ecological and the other economic. In terms of ecology, the geographic range of the three trees whose nuts are gathered extends throughout the Guiana Shield and the greater Amazonia region. While localised commercial extinction would be regrettable for the indigenous nut gatherers, *Carapa guianensis* would not be endangered globally. Similarly, the Trio of Sandlanding would have to find new *Ormosia costulata* trees if and when logging activities commence in their current collection sites. However, the O*. costulata* species are similarly not endangered in terms of their geographic range. We discovered no immediate ecological threats to the harvesting of seeds, as their collection did not involve cutting down trees and no other plants were destroyed in the collection sites. However, the long-term ecological impact from harvesting seeds from these tree species is currently unknown. As trophic nets are not well understood, disappearance of *Carapa* from the local forest ecosystem may have unknown ecological consequences, including on animals known to predate on leaves and fruits [39].

In the case of *Potamotrygon boesemani* harvesting, the economic returns on time spent in hunting were very high. As the *Potamotrygon boesemani* is endemic to the Corentyne watershed, localised extinction of the wild population here would signify total extinction of this wild population in its natural habitat. Future research should focus on determining and managing the ecological sustainability of NTFP extraction, following [40]: (1) quantitative resource inventory of targeted species, (2) growth, yield and regeneration studies; (3) assessment of harvest impacts; and (4) periodic monitoring and harvest adjustments. This collaborative research could be done to determine impacts on targeted NTFP species and their surrounding ecosystem. Local NTFP gatherers could be enlisted to do the monitoring for plant products, while hunters and fishermen could monitor wildlife populations.

### No distinct separation between ‘subsistence’ and ‘commercial’ offtake

None of the ITP communities of Suriname has State-recognised rights to either control or collaboratively manage the lands, territories and resources (LTR) that they depend on for subsistence or commercial sale. Furthermore, and critically, there is no clear line between ‘subsistence’ and ‘commercial’ offtake. At least a quarter of the total households in Apoera and three quarters in Sandlanding are almost totally dependent on the natural economy for their livelihoods; no member of those households gains a steady income from paid employment. This means that a large part of the income from sales of crabwood oil, gathered nuts, bush meat or fish is spent on food and other necessities for subsistence, health care or school supplies.

### Regulations and collaborative management strategies to deal with insecure tenure

The most important commercialised plant NTFPs—three varieties of nuts—are harvested outside the communal forest on ‘state domain’, or public lands that are zoned as State Production Forests (Table 1). In 2015, the communities had to relocate their crabwood seed harvesting because an airstrip was built on the main harvesting site. If logging concessions were to be granted over the area from which nuts and seeds are currently harvested, then the commercially desirable *Carapa* timber trees will be felled. In addition, road building and/or destructive or careless harvesting methods may threaten other tree species from which other NTFPs are gathered. The collection of *Bertholletia excelsa and Caryocar nuciferum* seeds are also currently carried out on an area of state domain that has been proposed to become a nature reserve. As noted earlier, commercial NTFP collection is prohibited within nature reserves. However, since the announcement of the proposed nature reserve in the 1980s, there have been no follow-up actions.

**Table 1:** Tenure and ecological issues related to the harvesting of commercially important NTFPs

Plant (p)/animal (a) NTFP	Family	Voucher specimen #	Bought by	Tenure issues	Ecological issues
Crabwood seeds *Carapa guianensis* L. (p)	Meliaceae	TvdB002	Local subsistence users Non-local subsistence users Non-local small-scale commercial users	Collecting on state domain under request for logging	Overharvesting seeds can decrease seedlings
Brazil nuts *Bertholletia excelsa* Bonpl. *(p)*	Lecythidaceae	–	Local subsistence users Local small-scale commercial users Non-local small-scale commercial users	Collecting happens on state domain, which is proposed to become a nature reserve	Overharvesting seeds for successive years can decrease seedlings of this species
Sawari nuts *Caryocar nuciferum* L. *(p)*	Caryocaraceae	–	Local subsistence users Local small-scale commercial users Non-local small-scale commercial users	Collecting happens on state domain, which is proposed to become a nature reserve	Overharvesting seeds for successive years, can decrease seedlings of this species
Kokriki seeds *Ormosia costulata* (Miq.) Kleinhoonte *(p)*	Fabaceae	TvdB020	Non-local subsistence users	Collecting happens on logging concession site	Non observed
Boesemani stingray *Potamotrygon boesemani (a)*	Potamotrygonidae	–	Non-local commercial users	Fishing happens on de facto open access river	Extreme decrease in population Potential threats to trophic system Potential ecosystem-wide issues
White-lipped peccary *Tayassu pecari (a)*	Tayassuidae	–	Local subsistence users Non-local subsistence users	Hunting happens on state domain and on logging concession sites	Decrease in population
Lowland paca *Cuniculus paca (a)*	Cuniculidae	–	Local subsistence users Non-local subsistence users	Hunting happens on state domain and on logging concession sites	Decrease in population
Wolf fish *Hoplias aimara (a)*	Erythrinidae	–	Local subsistence users non-local subsistence users	Non-observed	Decrease in population

The terms of natural resource concession licences could, as in other countries, prohibit loggers from felling or damaging any species on which local communities have high dependence. SBB can write in this restriction without the State admitting ITP resource rights. Likewise ‘Nature Reserve’ designation could admit a privilege to registered inhabitants of named villages to continue non-destructive collection of plant products for domestic use or commercial sale [41, 42].

### Collaborative management

With regard to wildlife, hunters reported decreases in population sizes for all commercially harvested species. Currently, national hunting regulations focus on bag limits—the number of species that can be carried in a bag on a single trip. Within this system, a hunter could theoretically make three trips a day during the entire hunting season, carrying the maximum number of hunted animals each time. To avoid such a perverse outcome—increasingly possible as natural resources concessions increase in number in Suriname, consequently raising the number of potential hunters—government and local community representatives might co-develop rules for sustainable off-take of the most important game species per season, rather than per trip. There are many replicable or adaptable examples from the US States and Canada of hunting rules, including tags issued for number of animals, tags or parts of tags to be retained until wildlife carcass is processed for sale, all tags and parts of tags to be returned to the issuing agencies for discharge and prevention of reuse [43, 44].

Monitoring and enforcement of NTFP extraction rules are a major challenge and often costly [45]. As there is currently no effective monitoring system in place, the success of any rules will depend on the willing participation of the local communities who have the greatest stake in the long-term sustainability of the natural resources on which they depend. The Surinamese authorities might consider adopting the tried and tested rule of subsidiarity in which decision-making is delegated to the lowest effective level. Subsidiarity is considered desirable in natural resources management where the target resources are important for local livelihoods [46]. Collaborative monitoring of tags by paid local community members, recruited as representatives of government agencies, should precede the setting of Annual Allowable Cut (AAC) rules and would allow communities to have active roles in setting fact-based rules and monitoring their execution.

In addition, if both governmental authorities and local community members were to collaboratively calculate the superior long-term value of gathered NTFPs versus the one-time payment from the sale of a *Carapa* log, for example, both parties might be galvanised to protect the nut-bearing trees. There are successful models detailing how this was done among Brazilian Amazonian communities [47–50]. Shanley’s research showed in addition the greater interest of women in sustainable production and stability of income from NTFP gathering and consequently the critical need to involve women, and not only men, in all discussions and rules related to collaborative management.

Turning to faunal offtake, our study has shown that the commercially most important fish—the endemic *Potamotrygon boesemani*—is harvested in the tributaries and main channel of the Corentyne River. While the Government of Suriname has total jurisdiction over this river which demarcates the international boundary with Guyana on the left bank, in practice the authorities only sporadically monitor the loggers, fishers and others from both Suriname and Guyana who traverse the river. The artisanal Guyanese fishers, who are primarily coastlanders [non-Indigenous], operate under the constant threat of piracy, with at least 14 deaths recorded in recent years [51]. The failure of the authorities to bring piracy under control is a telling indicator of the open access condition of the Corentyne River. The high prices paid for the *Potamotrygon boesemani* specimens in 2016 served as the trigger for its severe decline. Unsurprisingly, as our study showed, there were clear signs that the *Potamotrygon boesemani* population had been heavily reduced within a few months in 2016.

If better practices were followed for capturing and transporting these *Potamotrygon boesemani* specimens, many deaths could be avoided. Firstly, the stings of the ray should not be cut off but instead covered with a piece of plastic tubing to protect humans from stings and to safeguard the rays from infections. Secondly, fishermen and middlemen need proper tools to keep the water oxygenated and to lower the high mortality rates of captured rays during transportation.

In order to prevent this population from further catastrophic decline or even extinction, the Government of Suriname might consider placing an immediate moratorium on the export of *Potamotrygon boesemani*. Such a moratorium should be simultaneous with in-village trials of oxygenation systems, described below, to build confidence and acceptance. Consultations should take place with local communities to discuss and agree on specific steps to protect the remaining *P. boesemani* population. Inter-governmental discussions should also be held with the Guyanese governmental authorities and local communities, both to prevent stingray exports through Guyana and to develop a programme of collaborative management with the affected Guyanese communities. A moratorium would reduce the income of the local fishermen, which would cause dissatisfaction in the communities. However, the villagers have experience of the boom-and-bust cycles of other unregulated natural resources and are aware of the growing scarcity of *P. boesemani*. The continuation of the current unsustainable harvesting practices will soon result in zero stingray sales, which would be the most unwelcome outcome for both the communities and the *Potamotrygon boesemani* population.

A sustainable program could be set up to breed *Potamotrygon boesemani* in controlled environments within the local communities. *P. boesemani* are currently being bred in the Netherlands. Knowledgeable stingray breeders could be contracted to teach the indigenous villages how to set up water tanks with the necessary equipment and explain the best conditions for breeding [52]. In this way, the communities could still earn money through the sales of *P. boesemani*, while providing respite for the natural population to recover. As breeding programs have also been shown to be perfect fronts for the illegal trade of wild-caught specimens [53], the success of any such initiative is dependent on joint tagging, monitoring and reporting systems rather than on good faith compliance among all involved parties.

To monitor whether the *P. boesemani* population is resilient enough to make a comeback, research and monitoring systems should be put in place. Since long-term assessments are difficult to make in short-term scientific studies, monitoring can be done by the former harvesters [45], for which they should receive fair compensation. Besides keeping track of the natural population, further research should determine what the effects are on other natural elements of the Corentyne River basin.

These projects and studies could be funded by the government of Suriname which ratified the CBD. Article 10C requires State-Parties to: ‘Protect and encourage customary use of biological resources in accordance with traditional cultural practices that are compatible with conservation or sustainable use requirements’. The CBD definition of the terms ‘customary use’ and ‘traditional cultural practices’ refer to, inter alia, Indigenous legal systems for the control, use and management of land and natural resources. In order to comply with their obligations under Article 10C, States must ensure that national legislation and national policies account for and recognise, among others, Indigenous legal systems, corresponding systems of governance and administration, land and water rights and control over sacred and cultural sites [54].

If these recommendations regarding *Potamotrygon boesemani* conservation were followed, the government would fulfil its commitment to protect both biodiversity and indigenous practices. Local communities in West Suriname might develop a deeper bond with the *Potamotrygon boesemani*, the population would have a chance to recover and through breeding the species, and the communities could still earn an income. More broadly, collaborative management would build the necessary long-lasting relationships and agreements among local hunters and gatherers and governmental authorities on which sustainable natural resources management depend.

## Conclusions

This study addressed important issues regarding land tenure and harvesting rights in West Suriname. Firstly, the communal forestlands currently allocated by the Surinamese State to the Indigenous communities of West Suriname for customary uses do not correspond to their traditional and ancestral territory. Secondly, the Government-allocated lands are insufficient and do not meet the communities’ current needs and activities. Thirdly, the Surinamese State has not complied with the best practice guidelines set out in Article 10C of the Convention on Biological Diversity (CBD), which was ratified by Suriname.

All of the economically most important NTFPs are harvested outside the communal forest in lands over which the communities have no legal rights and only access rights. Consequently, local harvesters had to—and will again have to—relocate their NTFP gathering sites as a result of non-local or industrial developments. Therefore, there is an urgent need for collaborative management arrangements among government, industries and communities, mediated by FPIC procedures. Such a process would safeguard the local economy of the indigenous communities. We did not identify any current negative influence from harvesting plant NTFPs. However, the level of offtake of the endemic stingray *P. boesemani* is extremely unsustainable, and an immediate moratorium is needed in both Suriname and Guyana to prevent this species from being further imperilled.

## Figures and Tables

**Fig. 6:**
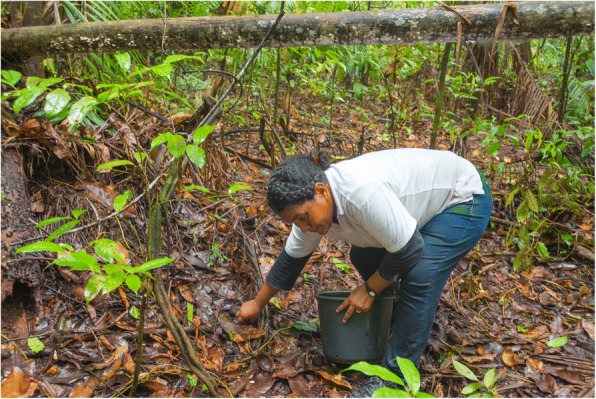
Crabwood seed collection. Marcelle Alpin collects crabwood seeds on the forest floor in the weekends

**Fig. 7:**
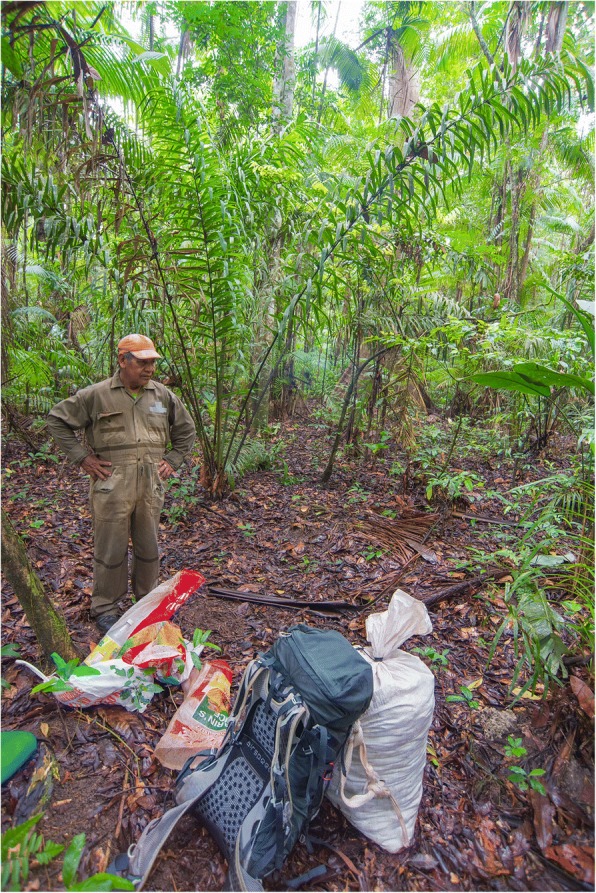
Crabwood seed harvest. Her husband Cyril Henry helps to gather seeds. The bags are completely filled with seeds and weigh over 50 kg

**Fig. 8:**
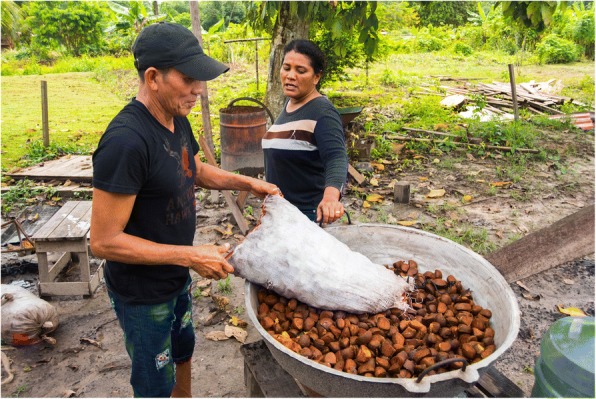
Prepare crabwood seeds for boiling. Julian helps Marcelle to load all the crabwood seeds into a huge bowl. After adding leaves from a certain plant, the seeds are ready to boil

**Fig. 9:**
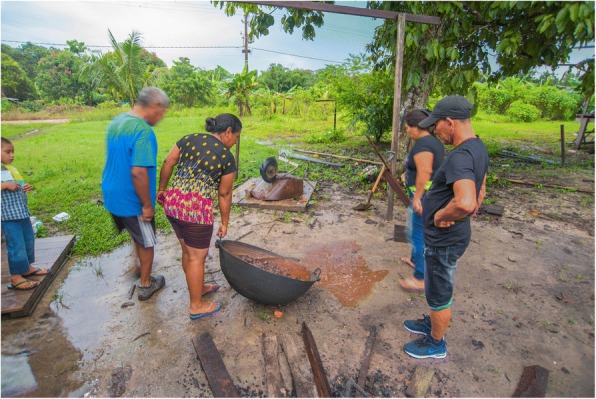
Prepare boiled seeds for drying. After approximately 6 h of boiling, the seeds are ready to be dried for about 2 weeks

**Fig. 10:**
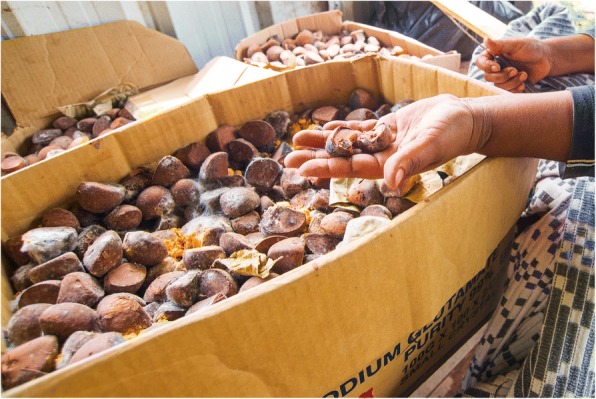
Fungal growth on dried crabwood seeds. When orange fungi appear on the dried seeds, they are ready to crack open with a knife, one by one

**Fig. 11:**
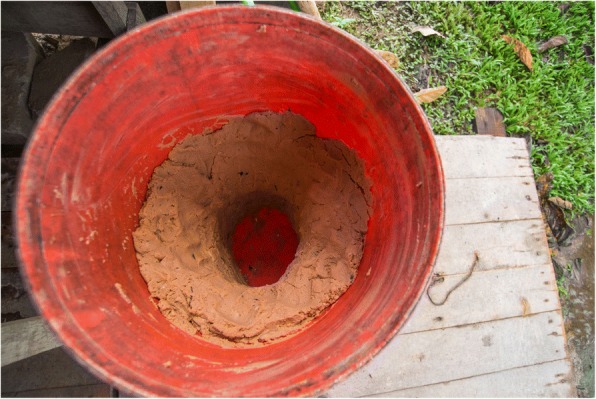
Paste from seed kernels. The inner paste from the cracked seeds is attached together and left to drain for about a week

**Fig. 12:**
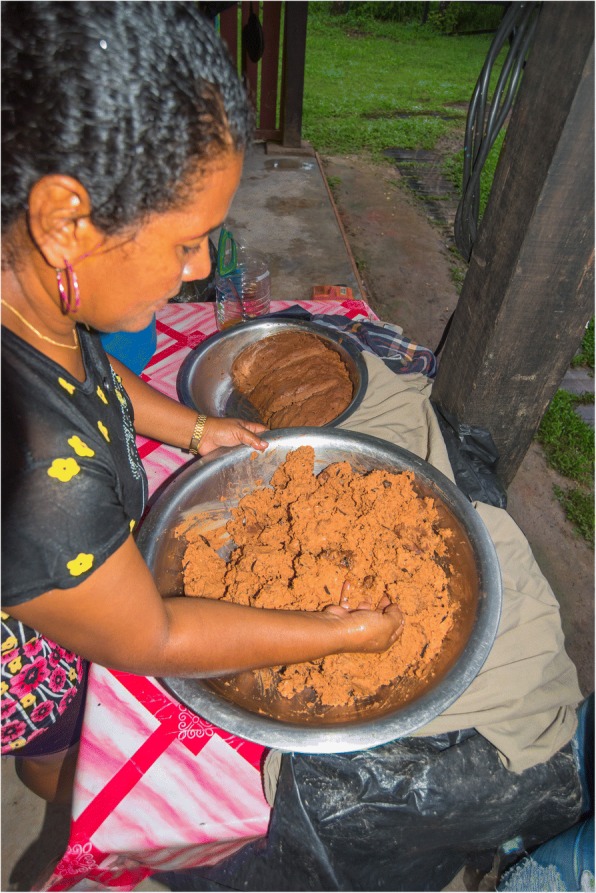
Manual kneading of kernel paste. The drained paste is then massaged three times per day for about 5 min

**Fig. 13:**
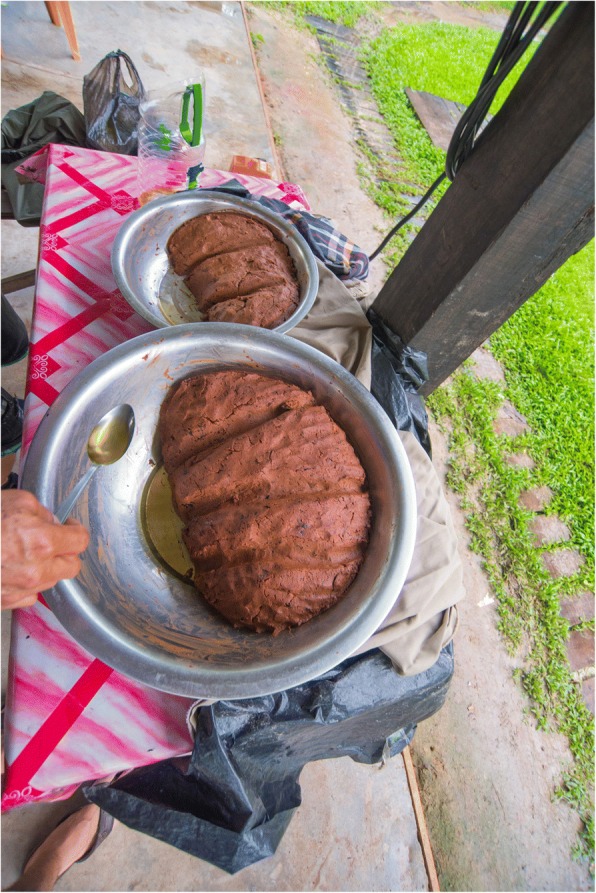
Separation of oil from crabwood kernel paste. The oil that has dripped out of the paste is removed and stored as crabwood oil

**Fig. 14:**
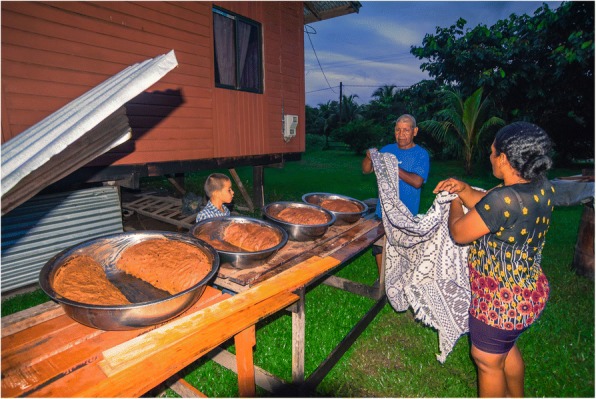
Crabwood kernel paste for oil production. The process of massaging is repeated for about 3 weeks until no more oil is appearing

## Appendix 1

**Table 2:** All commercialised NTFPs in the villages of Apoera and Sandlanding, West Suriname

Local name	English name	Scientific name	Family	Type	Voucher specimen #	Unit	Price per unit (US$)	Months sold	IUCN red list status
Krapa siri (Sr)	Crabwood oil	*Carapa guianensis* Aubl.	Meliaceae	Plant	TvdB002	Litre	5–7	Jul–Sept	Not listed
Ingi noto (Sr)	Brazil nut	*Bertholletia excelsa* Bonpl.	Lecythidaceae	Plant	–	Kilogramme	1.82	Feb–May	Not listed
Sawari (Sr)	Souari	*Caryocar nuciferum* L.	Caryocaraceae	Plant	–	Kilogramme		Jun–Jul	Not listed
Kokriki (Sr), weteu (Tr)		*Ormosia costulata* (Miq.) Kleinhoonte	Fabaceae	Plant	TvdB020	Piece of jewellery	1–10	Year round	Not listed
Spari (Sr)	Tiger stringray	*Potamotrygon boesemani* Carvalho & Almeida Wanderley	Potamotrygonidae	Fish	–	Animal	250	–	Not listed
Pingo (Sr)	White-lipped peccary	*Tayassu pecari* Link	Tayassuidae	Meat	–	Kilogramme	2.80	Aug–Mar	VU
Hei (Sr)	Lowland paca	*Cuniculus paca* Linnaeus	Cuniculidae	Meat	–	Kilogramme	2.80	Jan–Dec	LC
Anjumara (Sr)	Wolf fish	*Hoplias aimara* Valenciennes	Erythrinidae	Fish	–	Kilogramme	2.45	–	Not listed
Konkoni (Sr)	Agouti	*Dasyprocta leporina* Linnaeus	Dasyproctidae	Meat	–	Animal	2.80–5.60	Jan–Dec	LC
Marai (Sr)	Marail guan	*Penelope marail* Müller	Cracidae	Bird	–	Animal	2.80–5.60	Jul–Nov	Not listed
Pakira (Sr)	Collared peccary	*Pecari tajacu* Linnaeus	Tayassuidae	Meat	–	Kilogramme	4.90	Aug–Mar	LC
Dia (Sr)	Red brocket	*Mazama Americana* Erxleben	Cervidae	Meat	–	Kilogramme	2.60	Mar–Sept	DD
Sekrepatu (Sr)	Turtle	*Chelonoidis carbonaria* Bour	Testudinidae	Meat	–	Animal	5–10	–	Not listed
Kapuwa (Sr)	Capybara	*Hydrochaeris hydrochaeris* L.	Caviidae	Meat	–	Kilogramme	1.40	Jan–Dec	LC
Kapasi (Sr)	Armadillo	*Dasypus* sp.	Dasypodidae	Meat	–	Kilogramme	1.40	Aug–Mar	Not listed
Powisi (Sr)	Black currasow	*Crax alector* Linnaeus	Cracidae	Bird	–	Animal	8–14	Jul–Nov	VU
Bofru (Sr)	Lowland tapir	*Tapirus terrestris* Linnaeus	Tapiridae	Meat	–	Kilogramme	1.40	Jun–Aug	VU
Kubi (Sr)	Pacora	*Plagioscion surinamensis* Bleeker	Sciaenidae	Fish	–	Kilogramme	1.40	–	Not listed
Pacu (Sr)	Tambaqui	*Colossoma macropomum* Cuvier	Serrasalmidae	Fish	–	Kilogramme	3.50	–	Not listed
Mopé (Sr)	True yellow mombin	*Spondias mombin* L.	Anacardiaceae	Plant	–	Kilogramme	–	Feb–Jun	Not listed
Kujaridu (Tr)	–	–		Plant	–	Kilogramme	1.05	Jul	Not listed
Karau (Tr), switi bonki (Sr)	Ice cream-bean	*Inga* sp.	Fabaceae	Plant	–	Kilogramme	0.70	Jan–Mar	Not listed
Oloi (Tr), boskasju (Sr)	Kill my darling	*Dimorphandra conjugata* (Splitg.) Sandwith	Fabaceae	Plant	–	Kilogramme	–	Feb–Mar	Not listed
Groene boomboa (NL)	Emerald tree boa	*Corallus caninus* Linnaeus	Boidae	Animal	–	Animal	200	Jan–Dec	LC

*Sr* Sranang-tongo, *Tr* Trio, *NL* Dutch

## Appendix 2

The crabwood seeds are collected from the forest floor starting in March. Men help the women to collect the seeds and return the large bags, which sometimes weigh over 50 kg, back home. After the seeds have boiled for a couple of hours, they will be stored to dry. When red fungi start growing on the them after about 2 to 3 weeks, they are ready to be cracked open with a knife, one by one, in order to remove the inner seed paste. Then, the paste is left to drain for a week, after which it has to be massaged thrice a day. Finally, the oil will drip out over a period of another 3 weeks. These processes are constantly repeated until there are no more fresh seeds to be found on the forest floor. Crabwood oil is solely made by women.

## Abbreviations

AAC: Annual Allowable Cut
BBS: National Herbarium of Suriname
CBD: Convention on Biological Diversity
CIFOR: Center for International Forestry Research
CITES: Convention on International Trade in Endangered Trade of Wild Fauna and Flora
FAO: Food and Agriculture Organisation
FPIC: Free, Prior and Informed Consent
IFRI: International Forestry Resources and Institutions
ILO 169: International Labour Organization Convention 169
IPO: Indigenous Peoples Organisation
ITP: Indigenous and Tribal People
IUCN: International Union for Conservation of Nature
LTR: Lands, territories and resources
NTFP: Non-Timber Forest Product
SBB: Surinamese Forest Management and Production Control
TRAFFIC: Trade Record Analysis of Flora and Fauna in Commerce
UNDRIP: United Nations Declaration on the Rights of Indigenous Peoples
UN-FAO: United Nations Food and Agriculture Organisation
VIDS: Association of Indigenous Village Leaders (Vereniging Inheemse Dorpshoofden Suriname)
